# An Analysis of the Effects of In Vitro Photodynamic Therapy on Prostate Cancer Tissue by Histopathological Examination and Magnetic Resonance Imaging

**DOI:** 10.3390/ijms231911354

**Published:** 2022-09-26

**Authors:** David Aebisher, Michał Osuchowski, Dorota Bartusik-Aebisher, Magdalena Krupka-Olek, Klaudia Dynarowicz, Aleksandra Kawczyk-Krupka

**Affiliations:** 1 Department of Photomedicine and Physical Chemistry, Medical College of the University of Rzeszów, University of Rzeszów, 35-959 Rzeszów, Poland; 2 Medical College of the University of Rzeszów, University of Rzeszów, 35-959 Rzeszów, Poland; 3 Department of Biochemistry and General Chemistry, Medical College of the University of Rzeszów, 35-959 Rzeszów, Poland; 4 Center for Laser Diagnostics and Therapy, Department of Internal Medicine, Angiology and Physical Medicine, Medical University of Silesia in Katowice, 41-902 Bytom, Poland; 5 Center for Innovative Research in Medical and Natural Sciences, Medical College of the University of Rzeszów, 35-310 Rzeszów, Poland

**Keywords:** prostate cancer, photodynamic therapy, magnetic resonance imaging, ex vivo

## Abstract

Prostate cancer can significantly shorten the lifetime of a patient, even if he is diagnosed at an early stage. The development of minimally-invasive focal therapies such as photodynamic therapy to reduce the number of neoplastic cells while sparing delicate structures is extremely advantageous for treating prostate cancer. This study investigates the effect of photodynamic therapy performed in prostate tissue samples in vitro, using quantitative magnetic resonance imaging and histopathological analysis. Prostate tissue samples were treated with oxygenated solutions of Rose Bengal (RB) or protoporphyrin IX disodium salt (PpIX), illuminated with visible light, and then analyzed for changes in morphology by microscopy and by measurement of spin–lattice and spin–spin relaxation times at 1.5 Tesla. In the treated prostate tissue samples, histopathological images revealed chromatin condensation and swelling of the stroma, and in some cases, thrombotic necrosis and swelling of the stroma accompanied by pyknotic nuclei occurred. Several samples had protein fragments in the stroma. Magnetic resonance imaging of the treated prostate tissue samples revealed differences in the spin–lattice and spin–spin relaxation times prior to and post photodynamic action.

## 1. Introduction

**Figure 1:**
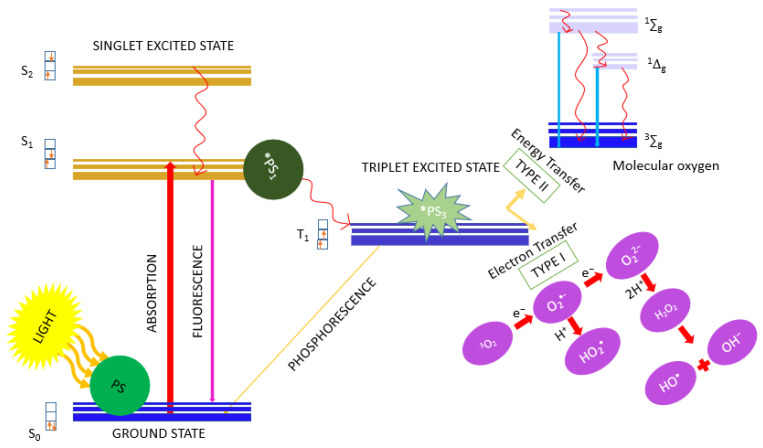
Type I and Type II pathways that elicit photodynamic action. The Jabłoński diagram above is a simplified graphical representation of Type I and Type II pathways that occur when a PS absorbs visible light. Singlet states are represented by S, and triplet states by T. The wavy arrows indicate internal conversion and intersystem crossing. Straight arrows illustrate transitions in which radiation is emitted or absorbed. S_0_—singlet ground state, S_1_—first singlet excited state, S_2_—second singlet excited state, PS—ground-state photosensitizer, *PS_1_—singlet excited-state photosensitizer, *PS_3_—triplet excited-state photosensitizer, ^1^Δ_g_—singlet oxygen (excited state), ^1^∑_g_—upper state excited, ^3^∑_g_—triplet oxygen (ground state).

Prostate cancer is the second most common malignant neoplasm in men [1,2,3,4,5,6,7]. According to the World Health Organization, prostate cancer had the highest incidence rate of 1 in 9.3 worldwide, assuming both sexes as the criterion and without the specified range of the criterion [8]. In prostate cancer, cell growth is a hormonal receptor-stimulated disease [9,10], and it is one of the few types of neoplasms for which no clear etiological factors can be indicated [11,12]. Histopathological examination is the basis for the diagnosis of prostate cancer, in which adenocarcinoma is mainly diagnosed. The acute and chronic forms of prostate disease include a complex set of diseases that can both lead to and result from cancer. Diseased states of the prostate require proper diagnosis and treatment, aided by introducing new focal therapies, such as photodynamic therapy (PDT), which is increasingly being applied as adjunctive therapy for cancer [13,14,15,16,17,18,19,20,21]. Deeper tumors are a challenge to treat by PDT due to the limited penetration of light into tissue. One of the conditions for carrying out PDT is the presence of a photosensitizer (PS) that elicits the cytotoxic photodynamic effect upon light absorption. Several photosensitizers have been approved for clinical PDT and photodynamic diagnosis (PDD), and they are often porphyrin or chlorin derivatives. Tissue-based photosensitizers (PS) concentrate in cancer tissue, and local exposure to a PS-infused region causes the production of reactive oxygen species (ROS), which destroys cells via apoptotic, paraptotic, or necrotic mechanisms (Figure 1). Singlet oxygen (^1^O_2_), generated by a Type II energy transfer from the excited triplet PS to ground-state oxygen, is believed to be the most important ROS in tissue-based PDT and vascular PS, such as the Tookad^®^ reactions via Type I processes, to generate hydroxyl radicals that damage tumor vasculature. Photodynamic therapy, used in prostate cancer, involves intravenous tissue or vascular PS injection, followed by a precise delivery of low-power laser light by optical fibers embedded in transparent plastic needles [22,23,24]. Due to the growing need to improve the diagnostic and therapeutic methods for prostate cancer (including PDT), in vitro experiments are useful. Currently, the development of PDT includes the synthesis of third-generation PSs that generate ROS as a result of near-infrared light absorption, lanthanide-doped upconversion nanoparticles that absorb infrared and emit visible light for deep tissue PDT, and the development of cell membrane-directed PS [25]. The in vitro results show that photosensitizers consistently and effectively reduce the viability of neoplastic cells [26,27].

Magnetic resonance imaging (MRI) can be used to detect the differences between healthy and diseased tissue, track the progression or disappearance of disease, monitor a course of treatment at the tissue level, locate neoplastic lesions, measure their distribution and size, and indicate surgical sites [28,29]. Magnetic resonance imaging (MRI) is commonly used to detect and characterize prostate cancer before and after PDT. It is challenging to test tissue samples ex vivo when only using standard coils with large surfaces and volumes. However, obtaining the relaxation times from individual measurements is extremely important and valuable in the context of comparing prostate cancer tissue samples before and after PDT [30,31]. The advantage of MRI is its excellent soft tissue contrast, which provides a powerful tool for delineating the prostate and prostate cancer. Furthermore, the 1.5-Tesla MRI has a high sensitivity for the detection of clinically significant prostate cancer and is routinely used in diagnostics [32,33]. In this study, we measured the spin–lattice (T_1_) and spin–spin (T_2_) relaxation times of prostate tissue samples prior to and post PDT. Spin–lattice mapping is a technique used to calculate the T_1_ time of a local region of tissue and display them as a parametric map. The spin–lattice relaxation time reflects the changes in intracellular and extracellular compartments and is affected by collagen, protein, water (edema), lipids and iron content [34]. The spin–spin relaxation time, also referred to as transverse relaxation, is a time constant for the decay of transverse magnetization and is tissue specific regarding its ability to differentiate healthy from diseased tissue [35]. This study attempted to evaluate the effect of photodynamic therapy on prostate cancer tissue ex vivo by combining the clinical MRI measurements of T_1_, T_2_, and by histopathological analyses before and after PDT.

## 2. Results

### 2.1. Relaxation Time Measurements

**Figure 2:**
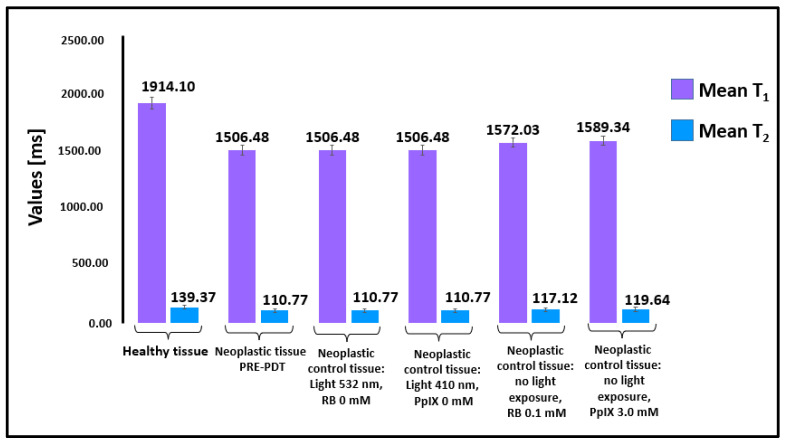
T_1_ and T_2_ relaxation times of healthy and neoplastic tissues (prior to the PDT procedure and control samples). Average T_1_ and T_2_ relaxation times between healthy (number of samples—65) and neoplastic prostate tissue (number of samples 65) were both statistically significant (*p* < 0.03). The number of samples in Figure 2 corresponds to the number of trials listed in Table 1.

The bar graph in Figure 2 shows the mean T_1_ and T_2_ values measured for healthy tissue and neoplastic tissue prior to the PDT procedure described in Section 4.3.

The difference in the means for the T_1_ and T_2_ relaxation times between healthy and neoplastic prostate tissue prior to the PDT procedure were both statistically significant (*p* < 0.03). For healthy tissue, the mean value for T_1_ was 1914.10 ± 52.43 ms, and 1506.48 ± 40.07 for the pre-PDT neoplastic tissue. For healthy tissue, the mean value for T_2_ was 139.37 ± 15.07 ms, and 110.77 ± 16.84 ms for the neoplastic pre-PDT tissue.

**Figure 3:**
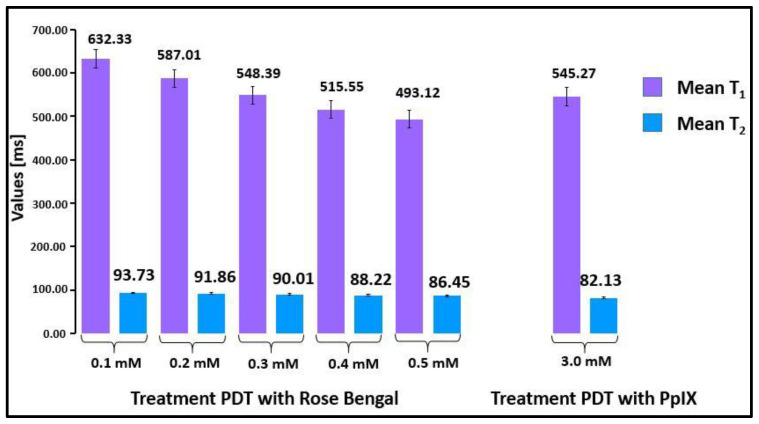
Mean values of T_1_ and T_2_ relaxation times for tissue treated with RB and PpIX. The difference in mean T_1_ and T_2_ relaxation times prior to and post PDT is statistically significant (*p* < 0.03). For comparison, the relaxation times measured for deionized water were T_1_ = 3245.45 ± 23 ms and T_2_ = 134.23 ± 11 ms. For 0.5 mM Rose Bengal, T_1_ = 2994.68 ± 10 ms and T_2_ = 129.32 ± 9 ms.

Figure 3 presents the compiled mean values of the T_1_ and T_2_ relaxation times after the PDT procedure on the neoplastic prostate tissue samples as a function of RB concentration and for 3 mM PpIX. We found that the differences in the T_1_ and T_2_ relaxation times of neoplastic tissue prior to and after the PDT procedure with RB were statistically significant (*p* < 0.03). Additionally, differences in the T_1_ and T_2_ relaxation times of neoplastic tissue prior to and after the PDT procedure with 3 mM PpIX were statistically significant (*p* < 0.03).

With an increase in the concentration of RB, the values of both relaxation times decreased. The measurement of relaxation times by clinical magnetic resonance imaging is a sufficient diagnostic tool for distinguishing neoplastic tissues before and after PDT.

### 2.2. Histopathological Analysis

**Figure 4:**
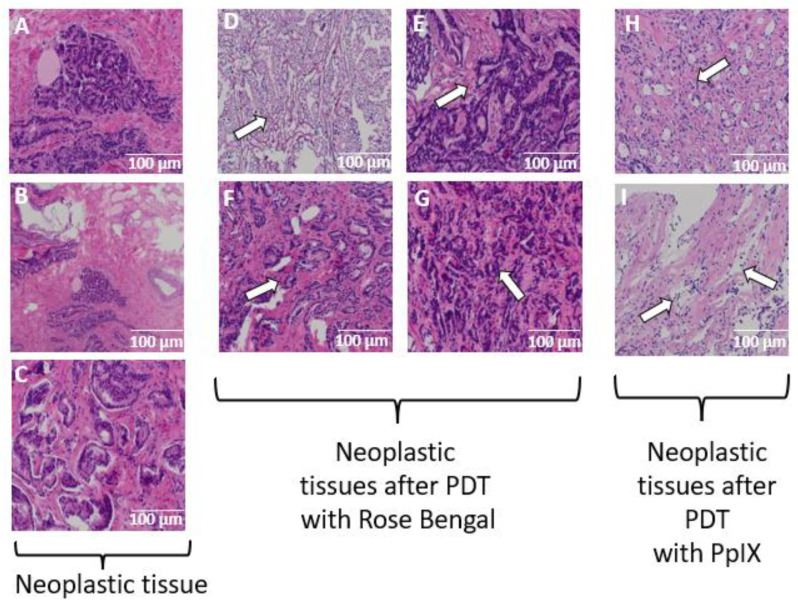
Prostate cancer tissue prior to (pictures **A**–**C**) and after the PDT procedure with (**D**) 0.2 mM RB, (**E**) 0.3 mM RB, (**F**) 0.4 mM RB, (**G**) 0.5 mM RB, (**H**,**I**) and 3 mM PpIX.

Figure 4 shows the microscopic images at magnifications of 63× and 100× for prostate cancer tissue after being subjected to the PDT procedure with RB and PpIX. As a result of exposure of the tissues to oxygenated photosensitizer solution and light, we detected an architectural deformation of the nucleoli and chromatin, along with edema and the appearance of protein fragments. The data indicate that RB and PpIX inhibit the viability of prostate cancer cells. The histopathology images of neoplastic tissue show a distinct tumor structure with visible nucleoli and chromatin. Tissue after the PDT procedure with 0.1 mM and 0.2 mM RB shows discrete chromatin condensation and moderate stroma edema. Tissue after PDT with 0.3 mM RB shows moderate to relatively massive and enhanced chromatin condensation with architectural disturbances of the nucleoli. Tissue after PDT with 0.4 mM RB shows small pycnotic nuclei along with severe architectural damage to the membrane. Moderate chromatin condensation is visible with well-marked stroma edema. It was nearly impossible to identify the phase of prostate cancer after PDT with 0.5 mM RB due to severe architectural disturbances. Massive chromatin condensation, pyknotic nuclei, and significant architectural disturbances were visible. Tissue after PDT with 3 mM PpIX revealed cell damage and necrosis features that are easily identified and enhanced. The number of cancer cells was reduced, and there was swelling (arrows in Figure 4D–I) in the stroma and a significant amount of protein fragments.

**Figure 5:**
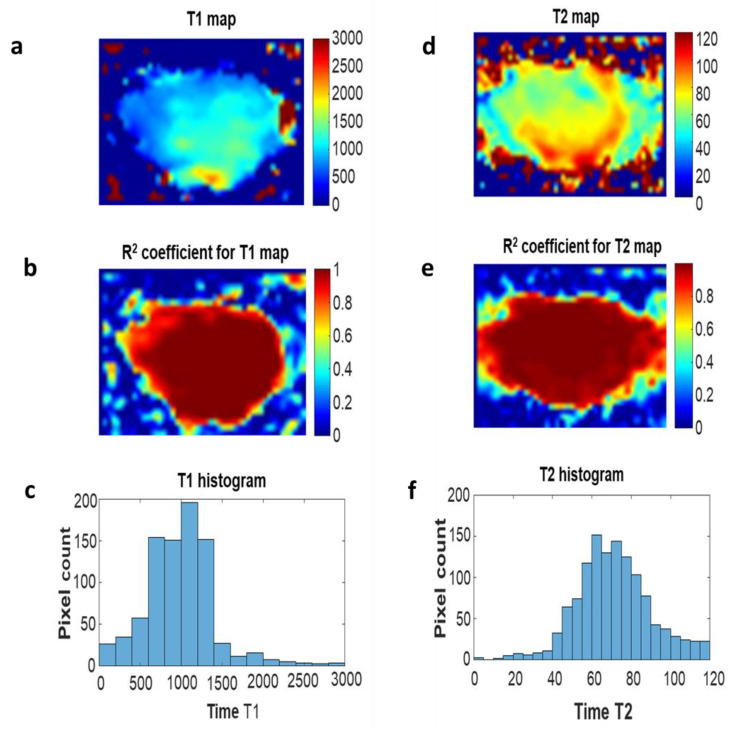
(**a**) Distribution of longitudinal relaxation time T_1_ values in the prostate tissue section after PDT with 0.4 mM RB; (**b**) fit factor R^2^ distribution; (**c**) a histogram of the values of T_1_ times determined from the image (**a**); (**d**) distribution of T_2_ transverse relaxation time values in the prostate cancer tissue section after PDT with 0.4 mM RB; (**e**) the distribution of the alignment factor, R2; (**f**) a histogram of the T_2_ time values determined from the image (**d**).

Figure 5 shows images from the histological analysis and T_1_ and T_2_ maps for the neoplastic tissue subjected to the PDT procedure using 0.4 mM.

## 3. Discussion

By analyzing the effects of the PDT procedure on prostate cancer tissue samples in vitro, we have determined that RB and PpIX have different effects on the tissue structure. The effectiveness of PDT in cancer tissue is believed to be associated with the aggregation of PS in cells. However, the uptake of PpIX and RB in the cellular environment is still poorly understood. This can be explained by the lack of an analytical method that allows for precise dosimetry of PpIX and RB in tissue. Here, we demonstrate that the combination of PDT, histology, and the relaxation time measurement is very robust for monitoring the photodynamic action in prostate tissue.

PDT is under investigation for a variety of applications in oncology. PDT for prostate cancer may have a role as an adjuvant local modality, especially in locations where the risk of failure is high. Additionally, PDT can be delivered at the time of surgical resection. The use of MRI for monitoring PDT treatment can be transferred to the clinic. According to the literature, PDT destroys the tumor by three mechanisms. The first mechanism is that ROS can kill cancer cells directly. The second mechanism is targeting the tumor vasculature, which obstructs the supply of oxygen and essential nutrients. A third mechanism occurs when the immune system is activated by PDT, triggering an inflammatory and immune response against cancer cells [36].

Cancer cells grow rapidly in an uncontrolled manner and have abnormal, disorganized vascularization with a defective inner lining. As a consequence, the tumor endothelium is leaky, and macromolecules may penetrate the extravascular space and be retained longer, compared to healthy tissues, due to impaired lymphatic drainage in the tumor tissue. This phenomenon is called the enhanced permeability and retention (EPR) effect and is often exploited for the treatment of cancer [37].

Additionally, the increased expression of some receptors on cancer cells helps to lower the pH inside the tumor and the number of macrophages that phagocytose the PS molecules [38]. Photodynamic therapy is a treatment that employs exogenously produced ROS to kill cancer cells generated from PS by light activation. Thus, the ROS generated by the PS is the key mechanism by which PDT elicits cell death and tissue destruction. The medical application of RB, a photosensitizer with high ROS generation capability, is limited due to its intrinsic toxicity and insufficient lipophilicity [39]. Protoporphyrin IX, induced by 5-ALA, promotes the generation of ROS and the induction of apoptosis via the activation of p53 and caspases in normal gastric cells but increases viability in gastric cancer cells. The molecular pathways involved in PpIX-induced cytotoxicity are not well-defined [40]. RB is reported to cause subcellular damage to the mitochondria, endoplasmic reticulum (ER), lysosomes, and the Golgi complex. Rose Bengal exerts long-term phototoxicity by activating both caspase-independent and dependent apoptotic pathways and autophagic cell death [41].

PpIX, a heme precursor, binds to the mitochondrial translocator protein and is transported to mitochondria to participate in heme metabolism. The application of porphyrin derivatives causes massive porphyrin accumulation in cancer cells [42,43,44]. In order to assess the effectiveness of PDT therapy in prostate cancer tissue in vitro, magnetic resonance imaging and microscopic examination of the histopathological specimens were used. Magnetic resonance imaging is able to distinguish the differences between tissue undergoing PDT therapy and healthy tissue. Submission of prostate cancer tissue to the PDT procedure generates a number of changes in the cellular and structural background due to the fact that the applied photosensitizer, through the accumulation in cells and exposure to laser light in the presence of oxygen, generates ROS, which directly or indirectly leads to cell apoptosis [45,46]. Photosensitizers are taken up by both healthy and diseased cells. In general, normal tissues are capable of eliminating or clearing a PS over time, while tumor tissues cannot do this due to non-existent lymphatics. This leads to some retention of PS in tumor tissue, which, when combined with localized activation light, gives PDT some additional selectivity. RB was shown to be toxic to cancer cells and to enter cancer preferentially, but not normal cells [47,48].

Prostate tissue, after extraction and freezing, does not change the morphology of cells. Many examinations, including those of the prostate, are performed intraoperatively and after the tumor is frozen. The histopathological examinations before and after freezing did not show any changes caused by freezing.

MRI can assist in the estimation of tumor volume. While MRI augments standard clinical and pathologic parameters in predicting advanced disease features and tumor volume, it is unable to reliably visualize small and well-differentiated cancers that may be prime candidates for either active surveillance or focal therapy. When it occasionally does highlight the area of a small, unifocal tumor, image-guided focal ablation may confidently target that region [49]. In 1980, the first endoscopic PDT procedure was performed for human lung cancer in a patient with poor cardiopulmonary function in whom surgery was not possible. This was an advanced squamous cell carcinoma obstructing the right main bronchus; PDT resulted in the opening of the bronchus. The second case was an early-stage squamous cell carcinoma of the right upper bronchus in March 1980 [50,51]. The variation in the initial tumor volumes (12–55 mm^3^) between different tumors within the same treatment condition could introduce variations in local tumor control [52]. Chang et al. discussed tumor size-dependent treatment with the use of ethyl pyropheophorbide a (MPPa) and N-methoxyl purpurinimide (NMPi) in an animal model, where high anticancer efficacy against small-size tumors was observed, indicating that early treatment with PDT is effective [53]. Small tumors (5–35 mm^3^) were found to respond well to a single round of PDT, while large tumors (35–65 mm^3^) showed no response to the same treatment [54]. PDT with 5-ALA was performed on 14 patients with histologically proven prostate cancer. The concentration of 5-ALA was 20 mg/kg body weight taken orally. A significant reduction in the PSA levels was observed 6 weeks after interstitial PDT [55].

As a result of the PDT procedure on prostate cancer tissues, there was chromatin condensation, stromal edema, nucleolus architectonic disorders, presence of trace protein, and necrotic cell damage. In a study by Wang et al., in histopathological preparations of prostate cancer in mice, it was observed that the nuclei of cancer cells treated with PSMA-1-PDT conjugates were significantly smaller compared to the untreated tumors. This directly indicated damage to the neoplastic cells with PDT [56]. In an in vivo study in a mouse model of prostate cancer, the tumor size was reduced due to PDT. Degeneration, an increased number of apoptotic cells and partial necrosis were observed. By histological analysis, Liu et al. showed necrosis of prostate cancer cells after PDT treatment with Nano-gel-Ce6-SAHA. Inflammation of the inflammatory cells in the area of necrosis was also observed [57]. Zaak et al. observed necrosis, a reduced number of live cancer cells and an area of apoptosis and necrosis with apoptotic cells in the central part [58]. All published studies confirmed the efficacy of PDT by analyzing histological slides. Assessing PDT by MRI is an innovative way to analyze the effectiveness of PDT. In this experiment, the tissues treated with PDT had lower T_1_ and T_2_ values compared to tissues before the PDT treatment. The mean value for T_1_ for the pre-PDT tissue was 1506.48 ± 40.07 ms and 629.31 ± 16.13 ms for the post-PDT tissue. The mean value of T_2_ for the pre-PDT tissue was 110.77 ± 16.84 ms and 93.23 ± 16.87 ms for the post-PDT tissue. In research conducted by Wang et al., preparations after PDT had different T_1_ and T_2_ values compared to the preparations which were tested before PDT [56]. Similar results were obtained by Fei et al. [30]. Both T_1_ and T_2_ relaxation times decreased following PDT of prostate tissue in vitro. This may be due to the loss of water from tissue, and this effect increased as the mass of the sample size decreased. This decrease in T_1_ and T_2_ is not expected to occur under in vivo conditions, where processes such as edema and increased hypoxia tend to increase T_1_ and T_2_. In a recent study, the T_2_ relaxation time measured following PDT of the brain in vivo [59] increased with a slight increase in T_1_, which was attributed to tissue edema and swelling. In these experiments, loss of water from tissue was responsible for the decreases in T_1_ and T_2_., In agreement with this work, the literature shows that the changes in the T_1_ and T_2_ values before and after PDT therapy are significant, which makes MRI a non-invasive imaging method for monitoring PDT-induced changes.

## 4. Materials and Methods

### 4.1. Prostate Tissue Samples

The prostate tissue samples were taken during prostatectomy at Clinical Hospital No. 1, Rzeszów, Poland. Immediately after removal, the prostate gland was evaluated in the Department of Pathomorphology, Clinical Hospital No. 1, Rzeszów, Poland. Tissues were collected from 30 patients diagnosed with advanced prostate cancer (well-differentiated prostate adenocarcinoma). As a result of the surgery, neoplastic tissue was removed along with a margin of healthy tissue. The healthy tissue margins that were collected served as controls in the experiments [60]. Controlled incisions were made, and a tissue fragment with hyperplasia features (healthy) and 2 or more tissue fragments suspected of a neoplastic lesion (diseased) were secured. Fragments with an average size of 8 × 4 × 4 mm^3^ were collected from the peripheral part of the organ (due to a much higher incidence of tumors in this area), and the second fragment was collected from the peripheral part of the organ with a high probability of benign nodular hypertrophy, with average dimensions of 5 × 3 × 2 mm^3^. The total samples collected from the 30 patients were 65 healthy and 65 neoplastic. Additionally, the healthy tissues were used as control samples. The remaining part of the prostate gland was fixed in formalin and subjected to standard histopathological evaluation. At the moment of acquiring the adenocarcinoma sample, the grade was not known but was determined later by a microscopic examination using the Gleason score. In this way, some samples were later identified as well-differentiated and some as poorly differentiated. For the PDT experiments, we chose samples with a Gleason score of 3 + 3 (grade 1) and 3 + 4 (grade 2) [61]. The secured tissue fragments intended for clinical trial were frozen in a cryostat at temperatures below −17 °C and then transported within 10 min to the tissue bank of the University of Rzeszów for storage at −72 °C. On the day of the experiment, the tissues were thawed to room temperature [62,63]. In all cases, we did not find extended extracellular spaces or shrunken cells resulting from the freeze–thaw cycle. These features are more pronounced in tissues stored for longer durations. The study was conducted in accordance with the Helsinki Declaration and approved by the Ethics Committee of the University of Rzeszów (protocol code 9/11/2018 and date of approval: 8 November 2018).

### 4.2. Photosensitizers

**Figure 6:**
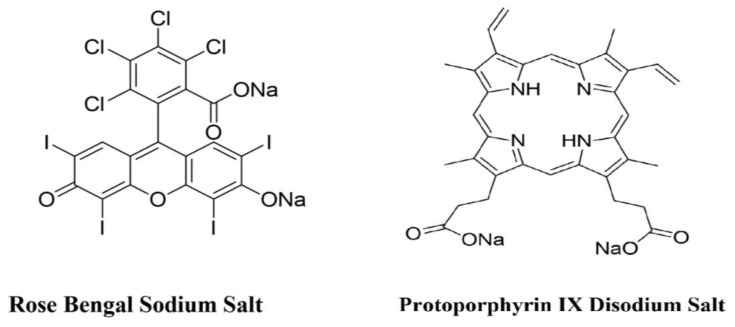
Structural formulas of photosensitizers (Rose Bengal Sodium Salt and Protoporohyrin IX Disodium Salt).

In this experiment, the following photosensitizers were used: Rose Bengal (RB) disodium salt (95%) (Sigma Aldrich, St. Louis, MO, USA) at a concentration of 0.1 mM (5 samples were charged with 0.1 mM RB without illumination, 6 samples of 0.1 mM RB with illumination), 0 mM (5 samples, illuminated), 0.2 mM (6 samples, illuminated), 0.3 mM (6 samples, illuminated), 0.4 mM (6 samples, illuminated), and 0.5 mM (6 samples, illuminated); Protoporphyrin IX disodium salt (PpIX) (Sigma Aldrich, St. Louis, MO, USA) was used for 15 samples at a concentration of 3.0 mM PpIX with illumination, 5 samples with 3.0 mM PpIX without illumination, and 5 samples with 0 mM PpIX with illumination. The structures of RB and PpIX are depicted in Figure 6. Water for the preparation of the photosensitizer stock solutions was purified with a reverse osmosis water treatment system (AquaB Duo, Fresenius Medical Care, Singapore). The stock solutions of RB and PpIX were purged with oxygen (99%, STP & DIN Chemicals, Bielsko-Biała, Poland) for 10 min prior to their addition to the prostate tissue samples. The number of samples and the concentrations of photosensitizers used in addition to the prostate tissue samples that were either irradiated or kept in the dark are presented in Table 1.

### 4.3. The PDT Procedure

**Figure 7:**
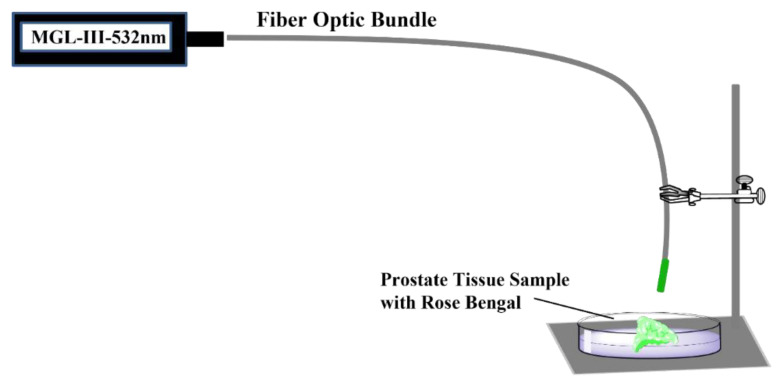
Experimental setup for illumination of RB-coated prostate tissue samples with an MGL-III 532 nm laser and Fiber Optic Bundle.

The prostate tissue samples were individually warmed to room temperature and placed in the center of a plastic Petri dish for the addition of oxygenated photosensitizer stock solution. The stock solutions of RB (0.1 mM–0.5 mM) or PpIX (3 mM) were purged with oxygen for 10 min. Immediately after oxygenation, a volume of 0.1 mL of a given stock solution was topically spread on the tissue dropwise, allowing the solution to cover the entire surface of the prostate tissue sample. The photosensitizer-coated tissues were then covered and kept in the dark for 1 h prior to irradiation to allow the PS to penetrate into the tissue [64,65]. After 1 h in the dark, the Petri dish containing the coated tissue samples was placed on a ring stand under a fiber optic cable that was connected to a solid-state laser via an SMA adapter and illuminated for 15 min. This distance of the light source from the tissue surface was selected to give an illumination area of 2.5 × 2.5 cm^2^, and the illumination of the samples did not cause the tissue to heat above 30 °C, as measured with a CPR-411 temperature probe (Elmetron, Zabrze, Poland). The apparatus for tissue illumination is depicted in Figure 7. For the illumination of the RB-treated samples, a solid-state laser (LD Pumped All-Solid-State Green Laser, MGL-III-532 nm/300 mW, Cni Laser, Changchun, China) provided 532 nm light, and the PpIX treated samples were illuminated at 410 nm (LD Pumped All-Solid-State Green Laser, MGL-III-410 nm/300 mW, Cni Laser, Changchun, China).

Immediately after 15 min of illumination, the tissue samples were cut in half, and one piece was sealed in an air-tight plastic tube for analysis by MRI, and the other was placed in a glass vial containing 10% buffered formalin solution (4% formaldehyde solution) for an eventual histopathological evaluation by microscopy. The control samples that were either treated with photosensitizer stock solution and not exposed to light or were exposed to light without photosensitizer were also divided into two pieces and stored in the same fashion.

Laser fluence was calculated using the formulas: energy [J] = power [W] × time [s], and fluence = energy [J]/area [cm^2^]. For example, 15 min (900 s) of irradiation on an area of 6.25 cm^2^ with a 300 mW laser gives a fluence of 43.25 J/cm^2^.

### 4.4. Analysis of Prostate Tissue Samples by MRI

The spin–lattice and spin–spin relaxation times of the prostate tissue samples that were sealed in an air-tight plastic tube from the PDT procedure were measured with a 1.5 Tesla Magnetic Resonance 360 spectrometer with a dedicated transmit–receive coil (General Electric Healthcare, Milwaukee, WI, USA). For the measurements of T_1_, the repetition time-TR was tested in the range of 50–15,000 ms by gradually increasing the time. There were 12 total trials. The value of the TR time was successively selected from the range of values: 50, 100, 200, 300, 500, 1000, 1500, 2000, 3000, 5000, 10,000 and 15,000 (all measured in a time unit equal to a millisecond. The technical parameters used in the MRI examinations were the same for all stages of the examination. The scan matrix was 256 × 256, and the field of view (FOV) was 6 cm × 6 cm with a section thickness of 1 mm, spacing of 0.5 mm and NEX = 2. The echo-TE time was constant and equal to 3 ms. Maneuvering the TR-time values made it possible to obtain the fast spin-echo (FSE) sequence, on the basis of which the MR images of the tested samples and their parameters were obtained. Fast spin-echo (FSE) imaging reduces the examination time and improves SNR. The test protocol for determining the T_1_ relaxation time consisted of the following steps: calibration of the system; recognition sequence—that is, locating an object in three planes (frontal, sagittal, transverse, 3-plane); coronal sequence (T_1_) of the fast spin-echo—FSE (with different values of the repetition time-TR).

For measuring the relaxation time T_2_, different spin-echo (TE) times were used. Different TE times (ranging from 1 ms to 250 ms) were used in 12 steps with the same scanning parameters, except for the repetition time, which was constant and amounted to 10,000 ms, and the echo time was in the range of 11.8–300 ms (11.8, 20, 42, 68, 85, 102, 130, 160, 200, 230, 250 ms). In this configuration, the repetition time was unchanged and amounted to 15 × 10^3^ ms. The next steps of the measurement were the same as for the T_1_ relaxation time (system calibration and recognition sequence), while step 3 was the T_2_ FSE frontal sequence, not the T_1_ FSE. MR scans were performed on pre-PDT and post-PDT tissues. The T_1_ values could then be computed pixel-wise from a signal intensity versus a time curve fitting model. The T_2_ values could then be computed pixel-wise from a signal intensity versus echo time curve fitting mode.

**Figure 8:**
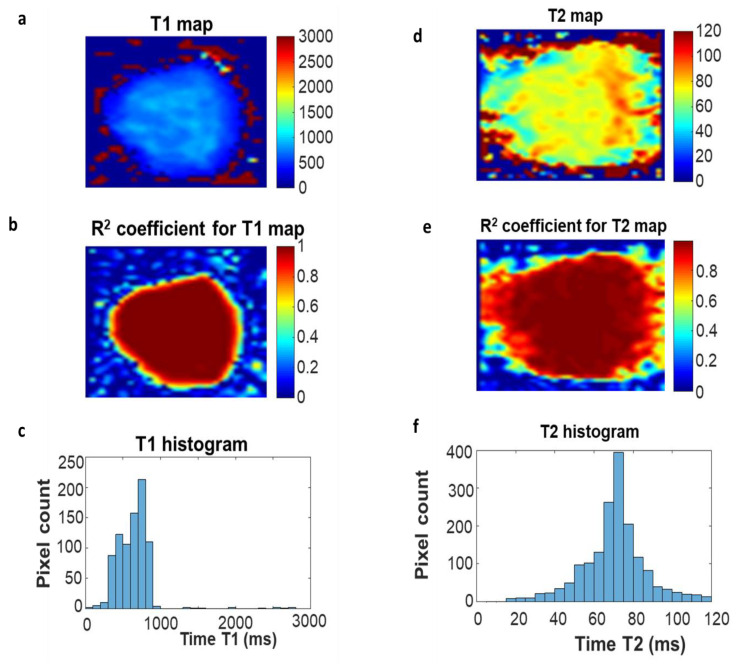
Selection of the area of interest for the measured samples: (**a**) distribution of the longitudinal relaxation time T_1_ in the prostate tissue section before PDT; (**b**) distribution of the R^2^ fit factor; (**c**) a histogram of the values of T_1_ times determined from image (**a**); (**d**) distribution of transverse relaxation time T_2_ values in a segment of the diseased tissue before PDT; (**e**) distribution of the R^2^ fit factor; (**f**) a histogram of the values of T_2_ times determined from the image (**d**).

A region of interest (ROI) was selected in the imaged tissues to calculate the T_1_ and T_2_ values. The region of interest measurements were selected very close to the tissues (Figure 8).

Postprocessing was performed using a GE workstation (AW_4. 6, General Electric Healthcare, Milwaukee, WI, USA). When measuring all samples, the ROI was drawn by hand. The histograms were obtained from the marked ROI areas. On the abscissa of these graphs, the values of the longitudinal and transverse relaxation time, respectively, were recorded, while the percentage value of the signal was recorded on the ordinate axis. General Electric software AV4.6 was implemented to perform a statistical analysis of T_1_ (at different TR values) and T_2_ (at different TE values); the average values of the T_1_ and transverse T_2_ magnetization relaxation times were obtained. The signal intensity measurements were performed in the default GE software. The measuring of the intensity of the measurement was used to determine the relaxation times. The measured signal intensity (SI) for each tissue (normal, pre-PDT, and post-PDT) within the region of interest (ROI) range was used to calculate the relaxation times, T_1_ and T_2_. These values were calculated in the software on the basis of the increase in the intensity of the magnetic resonance signal for T_1_ relaxation and the decay of the signal for T_2_ relaxation.

### 4.5. Statistical Significance

The data were analyzed using the Statistica 13.1 software (StatSoft Polska Sp.z o.o., Krakow, Poland). In order to compare the relaxation times, a student’s *t*-test for dependent samples was used to calculate the *p*-value parameter. Values were considered significantly significant when the *p*-values were ≤ 0.05.

### 4.6. Histopathological Preparations

Histological slides of the prostate tissue samples were prepared in the Clinical Department of Pathomorphology, Clinical Hospital No. 1. A tissue processor (Leica TP1020, Leica Biosystems, Deer Park, IL, USA), paraffin tissue embedder (Leica EG1150H, Leica Biosystems, Deer Park, IL, USA) and coverslipper (Leica CV5030, Leica Biosystems, Deer Park, IL, USA) were used.

The surgical material for the histopathological examinations was fixed for 24 h in a 10% buffered formalin solution (4% formaldehyde solution). After fixation of the prostate fragments, tissue sections were collected into cassettes. The tissue material from the cassettes was rinsed, dehydrated, passed through intermediate fluids and embedded in paraffin to obtain blocks. The sections were stained with hematoxylin and eosin. For this purpose, a universal device for staining the histopathological slides was used (Multistainer LEICA ST 5020, Leica Biosystems, Deer Park, IL, USA). The final step was to cover the sections with a coverslip; the space between the cover glass and the coverslip was filled with histofluid.

### 4.7. Microscopic Analysis

Histological image analysis was performed using a Leica DM1000 LED microscope (Leica DM1000 LED microscope, Leica Biosystems, Deer Park, IL, USA). The microscopic image unit was 100 µm. Microscopic images were obtained at magnifications of 40× and 100×.

## 5. Conclusions

The histopathological images of neoplastic prostate tissue samples subjected to the PDT procedure showed chromatin condensation, stromal edema and, in some cases, thrombotic necrosis accompanied by pyknotic nuclei occurred. Magnetic resonance imaging of prostate cancer tissue samples has been shown to be a helpful diagnostic tool in distinguishing pre- and post-PDT neoplastic tissue. Due to the fact that MRI provides information on the values of water relaxation times and their differences in healthy and neoplastic tissue, it is possible to assess the physico-chemical differences in tissues. The results of these experiments indicate the invaluable role of the usefulness of MRI relaxation times in tissue differentiation. The in vitro PDT therapy, using MRIs and histopathological analyses, enabled us to monitor the changes in neoplastic cells. It seems that the experiment may lead to further exploration of the in vitro and in vivo monitoring of PDT by MRI. Preclinical and clinical applications of PDT have shown promising results in prostate cancer. Photodynamic approaches used in prostate cancer treatment can have severe side effects, and it is necessary to improve the treatment by developing new PSs and dosimetric analyses.

## Figures and Tables

**Table 1:** Concentrations of photosensitizers used for PDT experiments, number of samples, and irradiation wavelength.

Prostate Tissue Sample Photosensitizer Concentrations			
Photosensitizer	Concentration [mM]	Wavelength of Light [nm]	Number of Samples
	0.1	control, no light exposure	5
Rose Bengal (RB)	control, 0 mM RB	532	5
	0.1	532	6
	0.2	532	6
	0.3	532	6
	0.4	532	6
	0.5	532	6
	3	control, no light exposure	5
Protoporphyrin IX disodium salt (PpIX)	control, 0 mM PpIX	410	5
	3	410	15

## Data Availability

The data presented in this study are available on request from the corresponding author. The data are not publicly available due to ethical issues.
